# Non-Oncological Radiotherapy: A Review of Modern Approaches

**DOI:** 10.3390/jpm12101677

**Published:** 2022-10-09

**Authors:** Valerio Nardone, Emma D’Ippolito, Roberta Grassi, Angelo Sangiovanni, Federico Gagliardi, Giuseppina De Marco, Vittorio Salvatore Menditti, Luca D’Ambrosio, Fabrizio Cioce, Luca Boldrini, Viola Salvestrini, Carlo Greco, Isacco Desideri, Francesca De Felice, Ida D’Onofrio, Roberto Grassi, Alfonso Reginelli, Salvatore Cappabianca

**Affiliations:** 1 Department of Precision Medicine, University of Campania “L. Vanvitelli”, 80138 Naples, Italy; 2 Radiation Oncology, Fondazione Policlinico Universitario A. Gemelli, IRCCS, Largo Agostino Gemelli, 00168 Rome, Italy; 3 Radiation Oncology, Azienda Ospedaliero-Universitaria Careggi, Department of Experimental and Clinical Biomedical Sciences, University of Florence, 50134 Florence, Italy; 4 Department of Radiation Oncology, Università Campus Bio-Medico di Roma, Fondazione Policlinico Universitario Campus Bio-Medico, Via Alvaro del Portillo, 00128 Rome, Italy; 5 Radiation Oncology, Policlinico Umberto I “Sapienza” University of Rome, Viale Regina Elena 326, 00161 Rome, Italy; 6 Radiation Oncology, Ospedale del Mare, ASL Napoli 1 Centro, 80147 Naples, Italy

**Keywords:** radiotherapy, non-malignant disorders, non-oncological radiotherapy

## Abstract

Despite being usually delivered in oncological patients, radiotherapy can be used as a successful treatment for several non-malignant disorders. Even though this use of radiotherapy has been scarcely investigated since the 1950s, more recent interest has actually shed the light on this approach. Thus, the aim of this narrative review is to analyze the applications of non-oncological radiotherapy in different disorders. Key references were derived from a PubMed query. Hand searching and clinicaltrials.gov were also used. This review contains a narrative report and a critical discussion of non-oncological radiotherapy approaches. In conclusion, non-oncological radiotherapy is a safe and efficacious approach to treat several disorders that needs to be further investigated and used in clinical practice.

## 1. Introduction

Radiation therapy (RT) represents one of the cornerstones of cancer management, together with surgery and systemic therapy. It is reported that almost half of all cancer patients will receive RT during their treatment [1].

In the last decades, RT has undergone several advances driven by the increase in knowledge of radiobiology, use of advanced imaging, and treatment delivery approaches [2,3,4,5]. 

Actually, RT can be delivered with great accuracy, to reach an increasing dose to the targets and at the same time sparing the surrounding organs at risk [2,3].

Non-oncological radiotherapy can be used to treat several disorders and accounts for 20% of all treated patients in Germany [6,7,8].

In many other countries, including Italy, the use of RT for non-oncological diseases is not very common among the RT centers and often unknown among the other specialists.

The knowledge and the promotion of non-oncological RT, thus, could counterbalance the expected loss of patients or RT fractions foregone in the next future due to the use of hypofractionation.

Herein, we will discuss the different non oncological diseases that can be treated by RT. Following a literature search, we will provide a narrative overview of these topics.

## 2. Search Strategy

A literature search was conducted to retrieve potential eligible studies using PubMed and the clinicaltrials.gov electronic database.

The literature search was performed in September 2022 (from 1980 to August 2022) using the keywords “Radiotherapy AND non oncological OR non malignant OR benign“.

Additionally, we manually searched the reference lists of studies and review papers to identify other relevant studies. No limits were applied to publication type. The results are grouped according to the non-oncological disorders and discussed qualitatively.

## 3. Results

### 3.1. Heart

Cardiac arrhythmias affected 8.8 million people in Europe and is an independent risk factor for stroke, [9], heart disease [10] and chronic kidney disease [11]. Recently, RT has been investigated as a potential treatment for recurrent ventricular tachycardia (VT) and atrial fibrillation (AF). The technological improvement of RT techniques might arguably open up to new treatments in this setting. We report the main data in the literature regarding RT treatment of VT and AF. A summary of collected evidence is reported in Table 1.

#### 3.1.1. Ventricular Tachycardia

Stereotactic radiotherapy (SBRT) is under investigation as a treatment option for patients with VT who do not respond to antiarrhythmic drugs and/or catheter ablation. The evidence is scarce, but most studies suggest the dose of 25 Gy in a single session.

Cuculich et al. combined techniques of electrocardiographic imaging to map arrhythmogenic scar regions in patients with refractory VT and non-invasive delivery of precise ablation with SBRT to perform noninvasive cardiac radioablation. SBRT dose was a single fraction of 25 Gy. Treatment efficacy was measured using the number of VT episodes recorded by ICDs [12]. Of the nine evaluated patients, only five underwent SBRT, with no acute high-grade toxicity or complications. Sixty percent of the patients showed mild fatigue and all the patients showed a reduction of VT [12]. Similarly, in the Polish SMART-VT Trial, Kurzelowski et al. used Stereotactic Arrhythmia Radioablation (STAR) for two patients with refractory VT, using the same dose (25 Gy in single fx). After 6 months, ICD showed no VT episodes in one patient and a good response in the other patient [13].

Wight et al., similarly, treated with the same dose 14 patients with refractory VT. In this trial, the clinical target volume was delineated according to the individual patient’s characteristics, based on electroanatomic mapping. Two patients died after SBRT, whereas one received a heart transplant, and another patient did not respond. Of the other 10 patients, VT was reduced in 59%, ATP was reduced in 39%, and shocks were reduced in 60% [14].

In the UK multicenter experience, seven patients were treated with the same dose and technique similarly to the previous experience. After 6 months, for the five patients analysed VT burden was reduced by 85%, with no high-grade acute toxicity and three deaths due to heart failure. [15].

The STRA-MI-VT was a phase Ib/II trial that evaluated the feasibility of Cyberknife tracking in treatment planning [16].

Bonaparte et al. [17] also performed a dosimetric analysis for Linac-based STAR for VT, using different treatment planning approaches. The authors concluded that among the different techniques and energies, the 10 MeV Flattening Filter Free (FFF) approach was the faster but not suitable in patient with cardiac implantable electronic devices [17].

A systematic review including 13 studies and 57 patients confirmed efficacy and safety of STAR for refractory VT/ventricular fibrillation (VF). Thirty-one patients (54%) had ischemic cardiomyopathy and fifty patients (88%) had prior catheter ablation (CA) for VT/VF. A single dose of 25 Gy was delivered to a mean PTV of 64.4 cc (range 3.5–238) with a mean safety margin of 3.3 mm (0–5). Electrical storm was shown in 7% after SABR. VT burden was reduced in all patients, but recurrence affected most of the patients (75%), with several adverse events (81%) and no treatment-related deaths. The authors concluded that STAR preliminary experience appears safe and efficacious despite the recurrence rate and deserve to be further investigated [18]. Despite this, there are still few conflicting data on the follow-up of patients and on the influence of respiratory movement on the cardiac dose. In fact, cardiac motion presents an important challenge because the VT isthmus is subject to both respiratory and cardiac motion. So, Akdag et al. provided first experimental evidence for real-time cardiorespiratory motion-mitigated MRI-guided STAR on the 1.5 T Unity MR-linac. A real-time cardiorespiratory motion-mitigated radiotherapy workflow was developed on the Unity MR-linac. A 15-beam intensity-modulated radiation therapy treatment plan (1 × 25 Gy) was created in Monaco v.5.40.01 (Elekta AB, Stockolm, Sweden) for the Quasar MRI^4D^phantom (ModusQA, Modus Medical Devices, London, Ontario, Canada). Simulations showed that cardiac motion decreased the target’s D98% dose between 0.1 and 1.3 Gy, with gating providing effective mitigation. So, real-time MRI-guided cardiorespiratory motion management greatly reduces motion-induced dosimetric uncertainty and warrants further research and development for potential future use in STAR aimed at simultaneously compensating cardiac and respiratory motions [19].

Kautzner et al. have published case series with the first postmortem immunohistochemical analysis of morphologic changes in the myocardium early and late after SBRT. The authors have found apoptosis followed by fibrosis that could explain the timing of the SBRT efficacy on VT [20].

In conclusion, STAR had reasonable VT suppression in patients where conventional treatment had failed.

#### 3.1.2. Atrial Fibrillation

AF affects about 40 million people in the world and increased the risk of stroke and heart failure. Current clinical management include antiarrhythmic therapy and eventually catheter ablation in drug refractory patients. Despite that, a subset of elderly patients are not responsive to systemic therapies and have an high risk of complications following catheter ablation, thus a non-invasive approach as STAR should be investigated. 

A systematic review collected available evidence (both preclinical and clinical setting) on the feasibility and efficacy of STAR, including photon RT (XRT) and particle beam therapy (PBT), in the treatment of AF. Twenty-one works (17 for XRT, 3 for PBT, 1 both) published between 2010 and 2021 were included. The main favorable finding consisted in the detection of electrical scar in 4/4 patients undergoing specific evaluation, whereas the minimum dose for efficacy was 25 Gy. No acute complications were observed below this dose and a great heterogeneity was observed among the included studies [22].

Di Monaco et al. performed a phase II trial that reported a preliminary experience of five patients treated for AF. The preliminary results showed no high-grade side effects and a good response in terms of AF control and no further use of antiarrhythmic drugs [21].

Ultimately, STAR represents a safe and effective non-invasive approach in the treatment of drug-refractory arrhythmias, but there is still poor data in the literature regarding long-term efficacy and follow-up. These two aspects, together with the choice of specific patient settings, represent the most important challenge to be investigated with well-defined studies.

### 3.2. Soft Tissue Disorders

Non-malignant, proliferative, soft tissue disorders are a very heterogeneous group of diseases, with a tumor-like phenotype, although they do not have malignant characteristics. These pathologies can reach large dimensions and cause serious organs involved deficits. Abnormal growth of fibroblasts or hypertrophic scar tissue (keloids), or inflammatory factors over-expression, such as bFGF, TGF-β, PDGF, EGF, and CTGF in Dupuytren’s disease, can be found at the basis of the onset of these disorders [23,24]. RT may play a role in these diseases control thanks to its anti-inflammatory, anti-proliferative and immunomodulatory effects, as well as has been confirmed by numerous reports, especially when conventional treatments have not achieved sufficient control of symptoms. We report the main data in the literature regarding RT treatment in keloids, Dupuytren’s disease, Peyronie’s disease, and fibromatosis.

#### 3.2.1. Keloids

Keloids are benign skin disorders based on excessive connective tissue proliferation during the normal scarring process. They typically appear after repeated surgeries at the same site, burns, after trauma, and deep dermis injuries. Pathogenetic mechanisms are not fully known, but the fibroblasts present in keloids have different characteristics compared to the fibroblasts present in normal skin [25]. Keloids are common, occurring in 5% to 15% of wounds and affecting both sexes equally. They mainly affect people 10 to 30 years old and are more commonly seen in those with family history of keloids [26]. The treatment of choice for keloids is surgery, which, however, shows 80% local recurrence; therefore, adjuvant RT can lower the risk of recurrence, as well as being the main treatment in cases of scar inoperability. Adjuvant RT can lower the risk of recurrence, as well as being the main treatment in cases of scar inoperability. Brachytherapy is effective to prevent keloid formation: the first session should start the same day as surgery and recommended doses are 5–6 Gy in three fractions or 5 Gy in four fractions [27,28]. Complete response rate of RT (either with electrons or orthovoltage techniques and brachytherapy) range from 50 to 98% according to the literature data (Table 2).

#### 3.2.2. Dupuytren’s Disease

Dupuytren’s disease is a fibrotic hyperplasia of connective tissue structures at the level of the finger band and palm. It is a rare condition, with a prevalence of about 2% and with a higher incidence in males (3:1 ratio). The pathogenesis is probably due to factors such as repeated trauma, alcohol and nicotine abuse, and hereditary factors. The diagnosis is purely clinical and sees around the 4th decade of life the presence of fibrotic nodules at the level of the hand that result in digito-palmar contracture leading to severe functional limitations. Dupuytren’s disease can be staged by Tubiana classification, which subdivides pathology according to symptomatology. The gold standard treatments for early stages are medical treatment with intralesional applications of xanthine oxidase/dehydrogenase inhibitor, allopurinol, or cytotoxic agents such as vinblastine or colchicine. Surgery is usually reserved for more advanced stages (Stage 3 Tubiana); the role of RT in the treatment of Dupuytren’s disease tends to be rather preventive and prophylactic than curative. Thus, the goal is to avoid future functional impairment and a future need for surgery [6]. The efficacy of RT is higher in early stages, inducing a significant reduction in fibroblast proliferation. RT is usually delivered with electron beam at 6MeV energy, while in other centers orthovoltage can be delivered alternatively. The volume treated includes palpable nodules with a safety margin of at least 10mm and the uninvolved structures are protected with lead-based shielding. Among the RT fractionations present in the literature, the most used, with better results both in terms of efficacy and limited toxicity, are the hypofractionations. The main results of the literature are shown in Table 2. Ledderhose disease is a rare type of plantar fibromatosis histologically related to Dupuytren’s disease, and it has been also effectively treated with RT [44]. 

#### 3.2.3. Peyronie’s Disease

Peyronie’s disease (PD) is a benign condition leading to plaque formation at the level of the tunica albuginea of the penis, leading to local pain and a change in curvature during erection. It usually affects men between the 4th and 6th decades of life with an incidence of 0.3–3%. The most likely mechanism is penile trauma causing inflammation of the tunica albuginea and eventually scarring with fibrotic plaque formation. The pathology presents with penile pain, plaque formation, deformity during erection, and subsequent erectile dysfunction. Diagnosis is clinical with identification and measurement of the plaque. Medical treatment involves the use of drugs for oral treatment such as vitamin E, tamoxifen, and colchicine and for intralesional injection verapamil and collagenase. If drug therapy fails, surgery remains the best option, especially in the cases of severe curvature or angulation and erectile dysfunction [6]. The use of RT is considered indicated in early-stage disease with soft, noncalcified plaques. RT techniques such as x-rays, photons, or low-energy electrons can be used; to have better dose distribution at the surface level, a bolus is also applied over the plaques. The target volume involves the entire plaque with a safety margin of 1 cm, protecting pubic hair, testes, and penile bulb with shielding. Irradiation can be in antero-posterior or latero-lateral projection, the latter with vertical fixation of the penis. The recommended schedule is single dose of 2–3 Gy for a total dose of 10–20 Gy. With this regard, Seegenschmiedt et al. focused their investigation on non-oncological RT publishing numerouscase series [7].

### 3.3. Muscle-Skeletal Disorders

Radiation therapy is consolidating over the years its role in the treatment of inflammatory or degenerative skeletal disease. Due to the control of pain, the two most frequent indications for radiotherapy in benign diseases are osteoarthritis and periarthritis. Moreover, RT is a non-invasive approach. Irradiation can be provided by LINAC or orthovoltage. A summary of the collected evidence is reported in Table 3.

#### 3.3.1. Osteoarthritis and Osteoarthrosis

Epicondylitis is commonly divided into two main branches: lateral, also commonly known as tennis elbow, and medial, the golfer’s elbow. Epicondylitis has a negative impact on patients quality of life with symptoms such as pain, joint mobility restriction, and even sometimes edema, limiting everyday activities and independence [55].

Hautmann et al. [45] irradiated 138 epicondylitis humeri, with linear accelerator, unlike most studies in the literature that used orthovoltage [46]. Patients had a median NRS (pain numeric rating scale) 7. Total dose was 6 Gy or 3 Gy (every other day). Between elbow treatments, 30% repeated RT for a partial or absent response. The authors reported a complete response of pain, NRS 0, with a follow-up at 24 months and demonstrated a comparable results of linear accelerator RT compared with orthovoltage. The same authors published in 2020 the results of reirradiation at a dose of 3 Gy (0.5 Gy/fx) and 6 Gy (1 Gy/fx), reporting a good control of pain at 24 months of 50.9% and a non-difference statically valid between the two dosages [47]. From the Micke et al. study, in addition, the effectiveness of low-dose RT using LINAC can be inferred compared to RT using orthovoltage. Both techniques manage to obtain an excellent control of the pain, especially in the long term. From the series, only the group of patients with gonarthrosis did not have a good response, probably due to the process of irreversible degeneration typical of this lesion [48].

Alvarez et al. [49] have treated 184 degenerative osteoarticular disorders with a dose of 6 Gy (1 Gy/fr every other day). Patients who did not benefit from the first round of RT repeated treatment, for a total dose of 12 Gy (52% of cases). Median follow-up was 8 months. Although 91% of patients reported improved pain, only 32.6% reported VAS pain 0–3.

The effectiveness of RT is to be found in the anti-inflammatory mechanism of low doses (0.5–1.5 Gy/fx), which determine inhibition of the interactions between leukocytes and endothelial cells, a decrease in the production of adhesion molecules to the endothelium, a decrease of mediators of inflammation, and less expression of pro-inflammatory cytokines. Of note, there is no guideline regarding regimen and dose fractionation, but the strategies have been determined empirically and it has been reported that various doses such as 0.5 Gy performed in six fractions had the same effect of a dose of 1 Gy in six fractions. The choice of fractionation is mostly based on in vitro experiments, which have shown that the anti-inflammatory effect of low doses RT was maximum at 48 h after irradiation, and it was lost after 72 h [56].

In the study of the Netherlands, there were reported negative results for RT, this time in the treatment of the knee osteoarthritis [50]. The authors conducted a randomized, double-blind, controlled study that showed that LDRT (6 Gy) does not lead to a substantial reduction of symptoms in patients with knee osteoarthritis. Note that unlike previous experiences, the follow-up in this study is relatively short and does not allow the long-term benefit to be assessed.

More recently, a prospective trial started in 2020 has been started by Niewald et al. including 236 patients (64 knees and 172 hands) all with a diagnosis of osteoarthritis (OA). The aim of this study was to compare two schemes of treatment (3 Gy vs. 0.3 Gy). The study showed no statistically difference in pain control between the two schemes. Further studies need to be performed because there’s no evidence about the effect of low doses such as 0.3 Gy in the literature [54]. 

Given the increasing interest in benign osteoarticular pathologies in recent years, Alvarez and colleagues have outlined a CT-based contouring atlas for non-malignant skeletal and soft tissue disorders [57]. The aim of the authors is to suggest a correct PTV delineation based on simulation CT, for treatment of painful shoulder syndrome, such as periarthritis humero-scapularis, epicondylitis humeri, finger joint osteoarthritis, trochanteric bursitis, gonarthrosis, plantar fasciitis, and Achiles tendinopathy.

#### 3.3.2. Achillodynia

Ott et al. in 2015 evaluated the long-term efficacy of two different RT schemes of 1 Gy/0.5 Gy over 3 weeks, twice per week used for 112 patients with diagnosis of achillodynia. The overall early (right at the end of RT), delayed (6 weeks after RT), and long-term (2 years after RT) response rates for all patients were 84 %, 88 %, and 95 %, respectively. This confirmed the positive role of RT for this kind of treatment [51]. In 2021, a review made by Rudat et al. analysed 666 patients for a total of 864 heels treated between 2009 and 2020. Since 2015, a questionnaire was given to all patients in order to measure the local pain control. For newly treated patients, after 3 months follow-up in case of an unsatisfactory pain control it was offered the possibility of a re-irradiation. Re-irradiation showed an improvement in local pain control of approximately 40%. More than 75% of the patients reported a good pain control, confirming the role of radiotherapy in this field [52]. This confirms the results of another study done by Hautmann in 2014 where 110 heel spur syndrome with lack of pain control has been re-irradiated and results were measured with NRS score system. Re-irradiation confirmed his role because 73.6% of the patients were free from pain 24 months after the treatment [53].

#### 3.3.3. Heterotopic Ossification

The role of RT on Heterotopic Ossification (HO) was supported by weak evidence. In 2014, a systematic review made by Ploumis et al. on a total of 27 studies of elbow HO showed that in most cases RT was stopped due to safety reasons. This review confirmed the lack of high-quality findings in the literature about RT in HO syndromes [58]. Thanks to the review made by Galietta et al. in 2022, it was finally confirmed the positive role of RT in the prevention of hip HO. Despite the numerous schemes available, from a single fractionated to several multiple fractionated schemes, no difference has been reported between the different schemes over the surgery alone [59].

### 3.4. Neurological Disorders

Radiation therapy has been widely adopted in the treatment of various neurological disorders, for different aims such as pain relief, control of the symptoms, and obliteration of brain arteriovenous malformations. Moreover, RT is a non-invasive approach that can be safely adopted also in elderly patients. A summary of the collected evidence is reported in Table 4.

#### 3.4.1. Epilepsy

A total of 0.5% of the world’s population suffers from epilepsy. About 30–40% of the patients do not benefit from pharmacological therapy and are eligible for surgical treatment. In patients where the pharmacological and surgical alternatives have been exhausted, RT is an option. RT, compared to surgery, has the advantage of not being invasive with low risk of neurological damage to the patient. Rauch C. et al. [60] reported first-time long-term outcome (median 10 years) of fractionated stereotactic radiotherapy (FSRT) in 11 patients with drug-resistant epilepsy. The biologically equivalent dose ranged from 26.3 to 58.3 Gy (α/β = 10). None of the patients developed temporary or permanent neurological deficits. Treatment resulted in improvement of seizure frequency in seven patients: five of them had a decrease in seizure frequency, and two of them were seizure-free at last follow-up. 

Liang S et al. [61] reported the long-term outcome of seven patients with temporal lobe epilepsy (TLE) treated with very low-dose LINAC based FSRT, treated with marginal dose of 12 Gy at the 85% isodose line. Reduction of seizure frequency post-FSRT was 50% in two cases, 30% in one case, and 0% in two cases, and seizure frequency increased more than 100% in two cases. No patient was seizure free at the last follow up. Two cases presented transitory complications and two cases showed an obvious drop in IQ, memory decline, and permanent neurologic complications, including partial aphasia and mild hemiplegia in one case, and progressive ataxia and cognition decline in another case. Bartolomei F et al. [62] reported outcome of 15 patients with TLE with median follow-up of 8 years (range 6–10 years) treated with gamma-knife with a marginal dose of 24 Gy. At the last follow-up, 9 of 16 patients (60%) were considered seizure free. A total of 60% of the patients experienced mild headache and were placed on corticosteroid treatment for a short period. All patients who were initially seizure free experienced a relapse of isolated aura (10/15, 66%) or complex partial seizures (10/15, 66%) during antiepileptic drug tapering. Restoration of treatment resulted in good control of seizures. Results are maintained over time with no additional side effects. Long-term results are comparable with conventional surgery.

Barbaro et al. [63] evaluated the effectiveness of radiosurgery, at the level of the amygdala, hippocampus, and parahippocampal gyrus as an alternative to surgery. Thirteen high-dose (24 Gy) and seventeen low-dose (20 Gy) patients were treated. Both groups showed significant reductions in seizures within a year of treatment, without observing major safety issues with high-dose stereotactic radiosurgery (SRS) versus low-dose SRS.

On these bases, radiotherapy has the potential to control the frequency and intensity of seizures in patients with pharmacoresistant epilepsy with mild long-term side effects if administered with proper fractionation, dose prescription, and target volume definition.

#### 3.4.2. Trigeminal Neuralgia

Trigeminal neuralgia (TN) is a neurological disease that cause intense facial pain, usually paroxysmal and excruciating, due to an alteration of the V cranial nerve (predominantly the mandibular and maxillary branches). In patients with medications failure/significant adverse events, radiotherapy (SRT, mainly SRS) may be an important therapeutic option. For SRS Gamma-Knife, linear accelerators and Cyber-Knife can be used. The three techniques showed no differences in terms of pain control, whereas the time to recurrence ranged from 6 to 48 months [78].

Smith et al. [64] performed a retrospective study that evaluated cohort of 169 patients treated with LIANC-SRS. The authors investigated different doses and volumes, concluding that increased dose and volume of brainstem irradiation improve clinical outcomes. Similarly, Rashid et al. [65] evaluated 55 patients with TN and treated with LINAC SRS up to a total dose of 90 Gy with 20% isodose line constraint to brainstem. After 30 months median follow-up, 69% of patients were pain free. Another important study has reported the outcomes of using Cyber-Knife SRS administered to 527 patients [66]. Dose prescription was 60–65 Gy to the 80–90% isodose line. A brainstem volume equal to or less than 1 cm^3^ was exposed at a dose of 10 Gy, with a maximum point dose (0.035 cm^3^) of 30% of the prescription dose. The pain relief rates were 87%, 82%, and 76% at 12, 24, and 36 months, respectively.

Lovo EE et al. [67] reported the outcome of 14 patients with TN treated with SRS to the Centromedian and Parafascicular Complex of the contralateral thalamus. They used a gamma angle regularly fixed at 90° and using a 4 mm collimator and the prescribed dose was 140 Gy to Dmax. Almost all patients (90%) reported some form of relief. Six patients (60%) reached the threshold of 50% pain relief. For four patients (40%), the procedure failed because the pain never improved.

Kundu B et al. [68] recently performed a retrospectively evaluation of patients with TN undergoing LINAC-SRS. The patients were evaluated with Barrow Neurological Institute (BNI) pain score and after a median follow-up of 5 months, 72% of the patients showed an improvement in this pain evaluation. 

A retrospective study by Fraioli et al. of 45 patients compared SRS to FSRT using a linear accelerator. The authors compared 40 Gy in a single fraction and 72 Gy in six fractions. Patients treated with FSRT showed a higher pain recurrence rate than SRS (27.3% versus 8.3%) [79].

Finally, reirradiation with SRS can still be used in case of recurrence, with a pain relief rate of 50% as showed in two retrospective studies [80,81]. The reirradiation was usually delivered between 15.7 and 26.1 months after the first irradiation, with a dose in the range 70–80 Gy.

#### 3.4.3. Brain Arteriovenous Malformations

Brain arteriovenous malformations (AVMs) are the persistence of a direct link between an artery and a vein; the nidus is located where small arteries and veins are connected [82]. Brain AVMs are rare (incidence estimated between 1.12 and 1.42 cases per 100,000 person-years) and mostly occur in young patients [83].

AVMs may be found as an incidental finding. They may be associated with intracranial hemorrhage, seizures, headaches, and/or neurological deficits. CT and MRI angiography are useful for the accurate diagnosis and nidus definition. Treatment approaches for AVMs are neurosurgery, embolization, and intracranial SRS. Stereotactic radiosurgery has a clinical obliteration rate of 60–80% [69,70,71,72]. The clinical benefit of SRS induced obliteration appear after 3 years or more. The success rate was increased with smaller volume (up to 30 cm^3^), lower Spetzler-Martin grade, higher dose, and steeper dose gradient. Embolization performed before SRS provided significantly lower obliteration rates than SRS alone (at 3 years: 41% versus 59%, respectively; *p* < 0.00001) [84,85]. Some AVM locations (functional areas, as thalamus) are related to poorer outcomes and require multimodal management [86]. Fractionated intracranial SBRT is poorly used in patients with AVM, mainly due to the low obliteration and morbidity rates initially reported [87]. Most recent results had an obliteration rate of 50% using fractionated intracranial SBRT delivering equivalent 2 Gy fraction doses higher than 70 Gy [88]. Symptomatic and permanent radiation-induced side effects have been described in 8 to 11% and in 1 to 4% of patients treated with SRS, respectively [71]. Stereotactic radiosurgery can be repeated when no complete obliteration is reported after the initial treatment. This is likely to occur in patients with AVMs larger than 10 cm^3^ and/or with high Spetzler-Martin grade [71]. Overall, the risks of hemorrhage and radionecrosis have to be considered and the treatment decision needs to be taken in a multidisciplinary setting in which the situation of each patient is assessed individually.

#### 3.4.4. Graves Ophthalmopathy

Graves ophthalmopathy (GO) is the most frequent extrathyroidal manifestation of Graves’ disease. Although GO is severe in only 3–5% of affected individuals, quality of life is severely impaired even in patients with mild GO. RT is a well-established method of treatment for GO. The main rationale is its anti-inflammatory effect and the high radiosensitivity of T lymphocytes and orbital fibroblasts. Although there are several reports about the benefits of RT [73,74,75], optimal initial treatment and its combination with steroids is still controversial. Various RT regimens with different doses and fractionations have been used: 16 or 20 Gy delivered in 8–10 fx (five days/week) is usually considered the standard [89]. A consensus statement from the European Group on Graves’ Orbitopathy does not recommend doses higher than 20 Gy [90]. Recent studies evaluated altered fractionation RT for GO. In a randomized study, Kahaly et al. [76] compared the efficacy and tolerability of three RT regimens of 1 Gy given weekly for 20 weeks for a total dose of 20 Gy; 1 Gy given daily for 2 weeks for a total dose of 10 Gy; and 2 Gy given daily for 2 weeks for a total dose of 20 Gy for patients with moderately severe GO. The authors concluded that whereas all regimens provided similar response rates, the protracted regimen had a better effectiveness and tolerance. Cordoso et al. [77] demonstrated the efficacy of orbital RT with a total dose of 10 Gy, fractionated in 1 Gy once a week over 10 weeks in 18 patients with GO.

Overall, favorable responses have been reported in 60% of cases. The best responses were noted for inflammatory signs and recent onset of extraocular muscle involvement. RT is well tolerated and safe and a careful selection of patients is necessary.

## 4. Discussion

All the above-mentioned disease can benefit from RT with interesting and promising results. At the same time, several pitfalls need to be managed before RT can be considered a safe and efficacious technique to manage non-oncological disorders.

Specifically, more work is needed in the field of radiobiology to correctly quantify the RT damage to organs at risk, especially in the risk of secondary cancers [91,92,93]. As the development of RT induced secondary malignancies represents one of the most important late side-effects, this topic has significantly influenced treatment decision-making and limited the use of RT for non-oncological disorders. Considering the increased life expectancy and the higher number of older patients, as well as the advanced in treatment delivery, all efforts should be made to prevent the incidence of tumors induced by radiation and to correctly estimate the individual risk.

At the same time, the number of prospective trials investigating the field of non-oncological RT is extremely poor and needs to be improved in the next years (see Table 5).

Most of the enrolling trials are investigating the new technique of Heart SBRT for arrhythmia disorders, whereas other diseases that can be managed with RT are not properly investigated. This aspect needs to be properly analyzed to test the role of modern RT in non-oncological fields and to sponsor the results among other specialists that do not know this approach. To this end, especially the scientific societies should undertake to study and spread the use of this type of RT, also involving specialists from other disciplines, following the path of DEGRO German Society.

In this context, it is noteworthy that the spread use of hypofractionation RT schedules is involving several cancer diseases and will decrease the number of fractions of patients at the LINACs [94,95,96] and so decreasing the reimbursements for RT centers. The knowledge and the use of non-oncological RT, thus, could counterbalance the expected loss of patients and fractions.

Scientific improvements of RT delivery, at the same time, needs to be tested in this particular field of disorders, as several other frontiers could be crossed in the next years that no-one can imagine now, such as the use of heart SBRT.

To the best of our knowledge, the present paper is one of the most recent overviews on RT for non-malignant diseases. Notwithstanding the narrative nature of the review, our results may strongly support the need for further investigation and represent a starting point for future clinical research, also in the context of RT Scientific Societies.

We finally recognize the limits of our review, in that it lacks explicit criteria for article selection and does not evaluate selected articles for validity.

By setting up the ambitious goal of addressing all these challenges, non-oncological RT can become a clinical reality in the next years and a higher number of prospective trials can be designed and conducted in the near future, with the endorsement of RT Scientific Societies. 

## Figures and Tables

**Table 1:** Summary of evidence regarding ventricular tachycardia (VT) and atrial fibrillation (AF) treatment with stereotactic ablative radiotherapy (SABR).

Authors	Year	N pts	Diagnosis	End-Point	Dose tot/fx	Results
Cuculich PS [12]	2017	5	VT	Efficacy and safety of treatment	25 Gy/1 fx	No complications during treatment. Fatigue after treatment (three patients), with no acute heart-failure. Marked reduction in the burden of ventricular tachycardia after treatment.
Kurzelowski R [13]	2022	2	VT	Efficacy and safety of treatment	25 Gy/1 fx	No problem in the first patient. The second one experienced acute side effects with an increase in VT that gradually improved at the end of the follow-up period.
Wight J [14]	2022	14	VT	Efficacy and safety of treatment	25 Gy/1 fx	VT was reduced in 59%, ATP was reduced in 39%, and shocks were reduced in 60%.
Lee J [15]	2021	7	VT	Reduction of VT and safety of treatment	25 Gy/1 fx	VT responded in all patients. After 6 months, VT burden was reduced by 85%. No high grade acute toxicity.
Piccolo C [16]	2022	Phantom study	VT	Feasibility of Cyberknife on cardiac lesions by tracking as a single marker the lead tip of an implantable cardioverter defibrillator.	25 Gy/1 fx	Tracking with a single marker is feasible considering adequate residual planning margins. The volumes could be further reduced by using additional markers.
Bonaparte I [17]	2021	Dosimetric study	VT	STAR is efficacy in terms of BDT and MUs.	25 Gy/1 fx	Several plans were evaluated for dosimetric considerations.
Kovacs B [18]	2021	57	VT/FA	STAR’s effectivity and safety for structural VT/VF	25 Gy/1 fx	Significant short-term reduction of sustained VT/VF-burden, but recurrences are common.
Akdag O [19]	2022	Phantom study	VT	First experimental evidence for real-time cardiorespiratory motion-mitigated MRI-guided STAR on the 1.5 T Unity MRlinac aimed at simultaneously compensating cardiac and respiratory motions.	25 Gy/1 fx	Cardiac motion was successfully mitigated using gating, which was demonstrated in the phantom and in-silico experiment.
Kautzner J [20]	2021	3	VT	postmortem immunohistochemical was performed early and late after SBRT	25 Gy/1 fx	Apoptosis and subsequent fibrosis was shown to be not immediate, thus the antiarrhythmic effects may be delayed after SBRT.
Di Monaco A [21]	2022	5	AF	Side effects at 1 month after STAR	25 Gy/1 fx25 Gy/1 fx	No acute treatment-related adverse events (>G1)

Abbreviation: N: number, Pts: patients, RT: radiation therapy, Fx: fractions, VT: Ventricular Tachycardia, AF Atrial Fibrillation, Gy: Gray, STAR: Stereotactic Arrhythmia Radioablation.

**Table 2:** Soft tissue disorders: included study and radiotherapy parameter.

Author	Year	N pts	Diagnosis	End Point	Dose	Results
Jiang [29]	2018	29	Keloids	Control rate	18 Gy/3 fx	Response rate 91.9%
Kim [30]	2015	28	Keloids	Control rate	12–15 Gy/3 fx	Response rate 50%
Shen [31]	2015	568	Keloids	Control rate	18 Gy/3 fx	Response rate 90.41%
Emad [32]	2010	26	Keloids	Control rate	12 Gy/3 fx	Response rate 70.4%
Malaker [33]	2004	64	Keloids	Control rate	37.5 Gy/5 fx	Response rate 97%
Lo [34]	1990	199	Keloids	Control rate	2–20 Gy/1 fx	Response rate 87% for Dose > 9 Gy, 43% for Dose < 9 Gy.
Borok [35]	1988	250	Keloids	Control rate	4–16 Gy/various fx	Response rate 98%
Van de Kar [36]	2007	21	Keloids	Control rate	12 Gy/3–4 fx	Response rate 71.9%
Arneja [37]	2008	25	Keloids	Control rate	HDR BT 5 Gy/3 fx	Response rate 92%
Van Leeuwen [38]	2014	67	Keloids	Control rate	HDR BT 6 Gy/2 fx	Response rate 96.9%
Jiang [39]	2016	32	Keloids	Control rate	HDR BT 6 Gy/3 fx	Response rate 94%
Hafkamp [40]	2017	29	Keloids	Control rate	HDR BT 13 Gy/1 fx	Response rate 75.9%
Kadhum [41]	2017	698	Dupuytren’s disease	Control rate	21–42 Gy in 3–14 fx	Good ratio of regressions (6–20% depending on staging), stability (12–81%) and low ratio of progressions (13–65%, depending on staging).
Seegenschmiedt [7]	2015	1762	Dupuytren’s disease	Control rate	15–21 Gy in 5–7 fx, 30 Gy split in 2 series of 5fx with a 3 months interval	Stability of disease in 84% for N stage and 67% for N/I stage
Betz [42]	2010	135	Dupuytren’s disease	Control rate	30 Gy split in 2 series of 5 fx separated by a 6- to 8-week interval	Stability of disease in 59%, 10% improved, and 31% progressed. In stage N 87% and in stage N/I 70% remained stable or regressed
Seegenschmiedt [8]	2015	8732	Peyronie’s disease	Pain, improvement	10–20 Gy (2–10 fx)	Pain regression in 50–90%, Improvement of penile deviation in 30–70%
Seinen [43]	2015	155 RT alone, 815 Surgery + RT	Fibromatosis	Local control	30–74 Gy	Local control in 78% of the patients treated with surgery and RT versus 85% in patients treated with RT alone

Abbreviation: N: number, Pts: patients, RT: radiation therapy, Fx: fractions, BT: brachytherapy.

**Table 3:** Muscle-skeletal disorders: included study and radiotherapy parameter.

Author	Year	N pts	Diagnosis	End Point	Dose	Results
Hautmann [45]	2019	124	epicondylitis humeri	pain relief	6 Gy(1 Gy)–3 Gy (0.5 Gy)	complete response 64% at 24 months
Rogers [46]	2020	157	epicondylitis, plantar fasciitis, and finger osteoarthritis	pain relief	4 Gy (0.5 Gy)–8 Gy Orthovoltage	pain relief at rest and during activity and a corresponding objective improvement in handgrip strength in epicondylitis. Pain relief at rest, during activity and improvement in walking time were demonstrated in plantar fasciitis
Hautmann [47]	2020	86	Humeral epicondylitis	pain relief	3 Gy/2.5 Gy (0.5 Gy/fx); 6 Gy (1 Gy/fx)	
Micke [48]	2018	703	Calcaneodynia, Achillodynia, Bursitis trochanterica, Shoulder Syndrome, Gonarthrosis	pain relief	6 Gy (0.5–1 Gy)	At follow up, good response: Calcaneodynia 80.7%, Achillodynia 88.9%, Bursitis trochanterica 46.3%, Shoulder Syndrome 60%; only Gonarthrosis 29.2%
Alvarez [49]	2019	108	OADD	pain relief	6 Gy (1 Gy)–12 Gy	Overall, and with a follow-up of 8 months (range 1–31 months), 91% of patients experienced pain relief. The pain reported according to the VAS scale was 0–3 in 32.6% of the patients, 4–6 in 36.7% and greater or equal to 7 in 20.1% of treated patients.
Mahler [50]	2018	55	knee osteoarthritis	pain relief	6 Gy	At 3 months follow-up: no substantial beneficial effect on symptoms and inflammatory signs of LDRT in patients knee OA, compared with sham treatment
Ott [51]	2015	112	Achillodynia	pain relief	6 Gy/3 Gy	Pain control:Early 84% Middle-term 88% Long-term 95%
Rudat [52]	2021	666	Heel Spur	pain relief	3 Gy (Re-irradiation possible)	Good local control (>75%) and good response to reirradiation
Hautmann [53]	2014	110	Heel Spur (Re-irradiation)	pain relief	3 Gy (Re-irradiation possible)	73.6% of Pain control after 24 months
Niewald [54]	2020	236	Kneel and Hand Osteoarthritis	pain relief	3 Gy/0.3 Gy	Good pain control with no difference between the two schemes

Abbreviation: N: number, Pts: patients, RT: radiation therapy, Fx: fractions, OADD: osteoarticular degenerative disorders, LCH: Langerhans cell histiocytosis, LDRT: low dose radiotherapy.

**Table 4:** Neurological disorders: included study and radiotherapy parameter.

Authors	Year	N pts	Diagnosis	End Point	Dose	Results
Rauch [60]	2012	11	Epilepsy	Tolerability and seizure frequency.	26.3–58.3 Gy	Treatment led to an improvement in the frequency of seizures in 63%.
Liang [61]	2010	7	Epilepsy	Seizure frequency	12 Gy	Reduction of seizure frequency was 50% in two cases, 30% in one case, and 0% in two cases, and seizure frequency increased more than 100% in two cases.
Bartolomei [62]	2008	15	Epilepsy	Seizure frequency	24 Gy	A total of 60% pts were considered seizure free. All patients who were initially seizure free experienced a relapse of isolated aura (66%) or complex partial seizures (66%) during antiepileptic drug tapering.
Barbaro [63]	2009	28	Epilepsy	Seizure frequency	24 Gy high dose vs. 20 Gy low dose	At the 36-month follow-up evaluation, 67% of patients were free of seizures for the prior 12 months (high dose: 10/13, 76.9%; low dose 10/17, 58.8%)
Smith [64]	2011	169	TN	Pain relief	70–85 Gy, 90 Gy	A total of 79.3% experienced significant relief. A total of 19.0% had recurrent pain. Of 87 patients with idiopathic TN without prior procedures, 79 (90.8%) had initial relief. Among 28 patients treated with 70 Gy, 18 patients (64.3%) had significant relief. Of the patients with 90 Gy at the brainstem, 59 (79.0%) had significant relief.
Rashid [65]	2018	55	TN	Pain relief	90 Gy	After 30 months median follow-up, 69% of patients were pain free.
Romanelli [66]	2019	387	TN	Pain relief	60 Gy (80% isodose)	Pain relief rate at 6, 12, 18, 24, 30, and 36 months was, respectively, 92, 87, 87, 82, 78, and 76%.
Lovo [67]	2019	14	TN	Pain relief	140 Gy	A total of 90% pts reported some form of relief. A total of 60% reached the threshold of 50% pain relief, and for 40% the pain never improved.
Kundu [68]	2022	41	TN	Pain relief	90 Gy	There has been a significant improvement in the post-radiation pain score in 72% of patients.
Starke [69]	2016	2236	AVM	Obliteration rate	20.5 Gy (mean margin dose)	Overall obliteration rate was 64.7%.
Ding [70]	2017	232	AVM	Obliteration rates, hemorrhage rate	22.5 Gy	The actuarial obliteration rates at 5 and 10 years were 72% and 87%, respectively. Annual post-SRS hemorrhage rate was 1.0%
Patibandla [71]	2017	233	AVM	Obliteration rates, hemorrhage rate in Grade III-IV AVMs	Mean dose 17.3 Gy	The actuarial obliteration rates at 3, 7, 10, and 12 years were 15%, 34%, 37%, and 42%, respectively. The annual post-SRS hemorrhage rate was 3.0%
Matsuo [72]	2014	51	AVM	Obliteration rate	15 Gy (80% isodose)	The actuarial obliteration rates at 3, 5, 10, and 15 years were 46.9%, 54.0%, 64.4%, and 68.0%, respectively
Matthiesen [73]	2012	211	GO	Symptomatic improvement	20 Gy/10 fx	A total of 84.2% pts reported a symptomatic improvement
Kouloulias [74]	2013	17	GO	Symptomatic improvementand tolerability	20 Gy/10 fx	Stabilization of the disease without recurrence was achieved in 12/17 patients. At the end of radiotherapy, the CAS regressed to 4.82 ± 2.24 (*p* < 0.001, Wilcoxon test). Extraocular motility and pain behind the globe were improved in 14/17 and 16/17 patients, respectively. Five patients developed recurrent signs and symptoms and they underwent surgical decompression
Li Yim [75]	2011	59	GO	duration of symptoms, clinical activity score (CAS)	20 Gy/12 fx (over 2 weeks)	Response (change in CAS) to orbital radiotherapy was statistically significant from 3.17 ± 1.75 standard deviation (SD) to 0.73 ± 0.92 SD (*p* < 0.001)
Kahaly [76]	2000	65	GO	Symptomatic improvement and toxicity	A: 20 Gy/20 fx (over 20 weeks) B: 10 Gy/10 fx (over 2 weeks) C: 20 Gy/10 fx (over 2 weeks)	Response to therapy, defined as a significant amelioration of three objective parameters, was noted in 12 A (67%), 13 B (59%), and 12 C (55%) subjects (C vs. A, *p* = 0.007). Ophthalmic symptoms and signs regressed most in group A
Cardoso [77]	2012	18	GO	Symptomatic improvement and Radiologic response	10 Gy/10 fx (over 10 weeks)	Significant decrease in symptoms such as tearing (*p* < 0.001), diplopia (*p* = 0.008), and conjunctival hyperemia (*p* = 0.002). Magnetic resonance imaging showed decrease in ocular muscle thickness and in the intensity of the T2 sequence signal in the majority of patients

Abbreviation: CAS: clinically activity score, SRS: stereotactic radiosurgery, AVM: arteriovenous malformation, GO: Graves ophthalmopathy, TN: Trigeminal neuralgia.

**Table 5:** Ongoing prospective clinical trial on non-oncological radiotherapy.

NCT Number	Disease	Design	Location
NCT04722263	Keloids	Single arm, interventional pilot study (15 patients). RT: 15 Gy in 3 fractions.	Montefiore Medical Center, New York, US
NCT04853433	Keloids	Single arm, interventional pilot study (15 patients). The primary endpoint will be toxicity.	Montefiore Medical Center, New York, US
NCT04122313	Dupuytren’s Disease	Prospective, Cohort study. Participants will be treated according to a standard treatment pathway, followed by post-operative radiation. RT: 15 Gy in 5 fx, followed by a 6–8 weeks break then a second identical course. Total dose: 30Gy.	University of Minnesota, US
NCT04424628	Gonarthrosis and Coxarthrosis	Non-inferiority study in which the investigators compare two low-dose radiotherapy schemes. Arm A will be treated at 3 Gy (0.5 Gy/fraction, 3 fractions/week), and patients in arm B will be treated at 6 Gy (1 Gy/fraction, 3 fractions/week).	GenesisCare, Malaga, Spain
NCT02708810	Trigeminal Neuralgia	To determine the feasibility of frameless Virtual Cone trigeminal neuralgia radiosurgery at a single institution prior to multi-institutional enrollment.	Hazelrig-Salter Radiation Oncology Center, Birmingham, Alabama, US
NCT03995823	Cerebral Arteriovenous Malformations	Prospective study including 50 patients with cerebral AVMs treated with GRKS to evaluate the sensitivity for nidus obliteration of MRI.	Department of Neurosurgery, Medical University of Vienna, Austria
NCT04843683	Cardiac Arrhythmias	Prospective, single-center, phase II trial that will be monitoring the safety and efficacy of using stereotactic ablative radiotherapy (SBRT) to treat arrhythmias.	University Health Network, Toronto, Canada
NCT04392193	Cardiac Arrhythmias	Proton Particle Therapy for Cardiac Arrhythmia Extracorporeal Energy Source Ablation of Cardiac Tissue: A First Stage Early Feasibility Study	Mayo Clinic, Rochester, Minnesota, US
NCT04984265	Cardiac Arrhythmias (Chagas)	SBRT in Chagas Disease Ventricular Tachycardia. A single 25 Gy dose will be delivered to the PTV.	University of Sao Paulo General Hospital, Sao Paulo, Brazil
NCT04642963	Cardiac Arrhythmias	Single arm, aimed at investigate safety requirements for clinical use. A single 25 Gy dose will be delivered to the PTV.	Medical University of Silesia, Katowice, Poland
NCT04833712	Cardiac Arrhythmias	The study aims to investigate the safety and preliminary efficacy of stereotactic radiotherapy for pulmonary vein isolation to treat refractory atrial fibrillation	Attikon University Hospital, Chaidari, Greece
NCT04486339	Cardiac Arrhythmias	Pulmonary Vein Isolation Using Stereotactic Radiotherapy System for the Treatment of Refractory Atrial Fibrillation	Xinhua Hospital, School of Medicine, Shanghai Jiao Tong University, Shangai, China
NCT03867747	Cardiac Arrhythmias	Radiosurgery for the Treatment of Refractory Ventricular Extrasystoles and Tachycardias (RAVENTA)	University Clinic Mannheim, Mannheim, Baden-Württemberg, Germany
NCT04162171	Cardiac Arrhythmias	Cohort Study—SBRT for VT Radioablation	Nova Scotia, Canada
NCT04066517	Cardiac Arrhythmias	STRA-MI-VT study is a spontaneous, open-label, not randomized, prospective clinical trial. The objective of the study is to evaluate the safety and efficacy of SBRT in strictly selected patients with refractory VT.	Istituto Europeo di Oncologia, IRCCS, Milan, Italy
NCT04612140	Cardiac Arrhythmias	Clinical trial: Patients with previously failed conventional RF catheter ablation will be randomized to radiosurgery (active treatment group) or repeated catheter ablation (control treatment group).	University Hospital Ostrava, Czechia

Abbreviations: SBRT: stereotactic body radiotherapy, RF: radiofrequency, VT: ventricular tachycardia, PTV: planning target volume, GRKS: gamma knife radiosurgery, AVM: Arteriovenous Malformations.

## Data Availability

Not applicable.
